# Supplementary material for: A Likelihood Approach for Real-Time Calibration of Stochastic Compartmental Epidemic Models
Source: PLoS Comput Biol. 2017 Jan 17;13(1):e1005257. doi: 10.1371/journal.pcbi.1005257 (PMC5240920; doi:10.1371/journal.pcbi.1005257)
Supplement: S1 File — (TAR.GZ) [file pcbi.1005257.s014.tar.gz › HSPH_Online-SI-Revision/PF08/n10-severe/n10-severe_PF_table4.pdf]

1	R0	{1.68952, 11.8402}	{1.87301, 19.8291}	{{1.76857, 19.3929}, {2.10963, 20.}}	{10.9592, 67.473}	inf	584.	1094.59	{1040.76, 1159.83}	87.4306	0.271904	newly	319.	363.88	{331., 395.}	14.3574	0.0575137	Reff	0.653675	0.699205	{0.608307, 0.760732}	10.0411	ar	6872.	9129.92	{8818., 9615.}	32.8568
2	R0	{1.71739, 9.62661}	{2.0557, 19.993}	{{1.93138, 20.}, {2.20578, 20.}}	{19.6992, 107.685}	inf	487.	1415.04	{1295.05, 1554.77}	190.562	0.462449	newly	307.	463.84	{423., 514.}	51.0879	0.178038	Reff	0.669954	0.665195	{0.605515, 0.723259}	6.08633	ar	6874.	9785.77	{9460., 10035.}	42.3592
3	R0	{1.48936, 6.23975}	{1.92966, 15.979}	{{1.71153, 12.2105}, {2.36788, 20.}}	{29.5632, 156.084}	inf	196.	1078.32	{681.07, 1537.17}	450.162	0.716997	newly	176.	432.3	{344., 519.}	145.625	0.384989	Reff	0.69285	0.725261	{0.620001, 0.791159}	8.82709	ar	5666.	9486.17	{8756., 10553.}	67.4227
4	R0	{1.62394, 7.51135}	{1.7479, 19.9521}	{{1.71352, 19.8603}, {1.77971, 20.}}	{7.63328, 165.626}	inf	314.	1044.61	{1009.94, 1079.03}	232.679	0.521811	newly	272.	348.74	{329., 365.}	28.2132	0.107459	Reff	0.684491	0.718789	{0.694225, 0.737794}	5.02345	ar	6417.	8621.72	{8439., 8788.}	34.3575
5	R0	{1.53632, 8.57652}	{1.76023, 19.9393}	{{1.69449, 19.7672}, {1.81499, 20.}}	{14.5745, 132.487}	inf	366.	1082.05	{1009.05, 1136.19}	195.643	0.470328	newly	274.	361.01	{330., 387.}	31.7555	0.118998	Reff	0.697643	0.722676	{0.682545, 0.764616}	5.37878	ar	6260.	8871.18	{8630., 9061.}	41.7121
6	R0	{1.47166, 5.7434}	{1.76481, 19.8427}	{{1.71516, 19.3708}, {1.85319, 20.}}	{19.9198, 245.487}	inf	206.	1080.15	{1017.62, 1118.2}	424.345	0.719391	newly	216.	363.27	{331., 387.}	68.1806	0.225109	Reff	0.704484	0.779796	{0.736958, 0.812482}	10.9357	ar	5650.	8515.69	{8307., 8703.}	50.7202
7	R0	{1.48179, 8.87369}	{1.64882, 19.0088}	{{1.50522, 14.2119}, {1.79372, 20.}}	{11.3414, 114.215}	inf	206.	829.925	{493.278, 970.856}	302.876	0.593638	newly	142.	288.43	{221., 330.}	103.12	0.302922	Reff	0.774828	0.742984	{0.690077, 0.777444}	5.7448	ar	5233.	8027.6	{7294., 8511.}	53.4034
8	R0	{1.53669, 6.47807}	{2.07082, 19.2573}	{{1.853, 17.1083}, {2.22507, 20.}}	{34.7583, 197.269}	inf	235.	1335.48	{1025.06, 1475.3}	468.291	0.750377	newly	203.	449.83	{405., 488.}	121.591	0.344303	Reff	0.682444	0.651467	{0.568954, 0.715865}	7.01962	ar	5934.	9327.68	{8761., 9673.}	57.1904
9	R0	{1.57227, 6.20989}	{2.07245, 15.7813}	{{1.7124, 10.4875}, {2.38227, 20.}}	{31.8125, 154.131}	inf	357.	1412.52	{823.488, 1975.87}	295.664	0.576252	newly	361.	569.61	{473., 650.}	57.7867	0.195105	Reff	0.71271	0.68378	{0.628723, 0.736202}	5.78846	ar	6136.	9316.81	{8212., 10076.}	51.8385
10	R0	{1.54936, 6.50938}	{2.01887, 13.9658}	{{1.66942, 8.97176}, {2.42951, 20.}}	{30.3034, 114.55}	inf	238.	1059.36	{591.179, 1703.92}	345.109	0.618124	newly	209.	471.24	{404., 547.}	125.474	0.350463	Reff	0.623308	0.664832	{0.540749, 0.734888}	11.4288	ar	6275.	9474.39	{8427., 10612.}	50.9863
11	R0	{1.42179, 3.18601}	{2.23597, 17.6536}	{{2.03534, 15.1663}, {2.4546, 20.}}	{57.2645, 454.097}	inf	53.	1498.22	{1194.93, 1783.}	2726.83	1.44482	newly	90.	544.41	{490., 598.}	504.9	0.780418	Reff	0.684876	0.623253	{0.593022, 0.661365}	9.39729	ar	5322.	10163.1	{9708., 10578.}	90.9637
12	R0	{1.48035, 6.46183}	{2.19912, 18.7242}	{{1.82963, 16.1813}, {2.39253, 20.}}	{48.5682, 189.767}	inf	313.	1588.69	{1358.45, 1735.39}	407.569	0.700052	newly	284.	550.84	{489., 612.}	93.9577	0.28576	Reff	0.695468	0.651231	{0.584573, 0.775458}	11.0169	ar	5793.	9759.85	{9008., 10183.}	68.4766
13	R0	{1.75755, 8.53854}	{2.21161, 19.9566}	{{2.16665, 20.}, {2.26117, 20.}}	{25.8348, 133.723}	inf	415.	1445.25	{1343.74, 1518.8}	248.253	0.541407	newly	276.	471.47	{440., 505.}	70.8225	0.231911	Reff	0.648009	0.614532	{0.569332, 0.652166}	6.30516	ar	6832.	9878.45	{9730., 10012.}	44.5909
14	R0	{1.47355, 4.89451}	{2.24646, 18.2678}	{{1.81675, 16.1899}, {2.4137, 20.}}	{52.4522, 273.231}	inf	140.	1205.77	{1030.74, 1420.65}	761.264	0.929769	newly	163.	423.6	{371., 481.}	159.877	0.412911	Reff	0.670023	0.572471	{0.506628, 0.691839}	16.2625	ar	5710.	9753.87	{8866., 10141.}	70.8208
15	R0	{1.62071, 5.98665}	{2.24299, 13.2826}	{{1.81891, 8.48832}, {3., 20.}}	{38.3952, 121.871}	inf	218.	1103.55	{552.524, 1850.01}	406.215	0.65034	newly	197.	489.5	{376., 594.}	148.477	0.38836	Reff	0.578593	0.577861	{0.407426, 0.685659}	16.0713	ar	6635.	9855.04	{8876., 11084.}	48.5311
16	R0	{1.67268, 7.7256}	{2.04462, 14.3242}	{{1.78308, 11.4431}, {2.58518, 20.}}	{22.2364, 85.4126}	inf	248.	851.266	{593.387, 1290.8}	243.252	0.511777	newly	184.	368.43	{316., 427.}	100.234	0.298031	Reff	0.552988	0.591113	{0.458143, 0.655768}	14.1418	ar	6947.	9508.79	{8850., 10665.}	36.8762
17	R0	{1.67805, 11.1417}	{1.96469, 18.7972}	{{1.75181, 14.9979}, {2.16362, 20.}}	{17.0817, 68.7104}	inf	598.	1388.54	{979.857, 1571.95}	132.197	0.359769	newly	353.	485.72	{436., 527.}	37.5977	0.137453	Reff	0.775091	0.752591	{0.69827, 0.794574}	4.18532	ar	6625.	8896.92	{8224., 9352.}	34.2931
18	R0	{1.66743, 9.99046}	{2.0713, 19.7268}	{{1.87431, 20.}, {2.23735, 20.}}	{24.2212, 97.4565}	inf	590.	1415.04	{1323.92, 1529.42}	139.838	0.377348	newly	394.	472.34	{429., 514.}	20.401	0.0796545	Reff	0.682146	0.690235	{0.622992, 0.756563}	5.95758	ar	6899.	9456.04	{9033., 9790.}	37.0639
19	R0	{1.70787, 9.85478}	{1.81567, 19.982}	{{1.78741, 20.}, {1.84966, 20.}}	{6.48571, 102.765}	inf	473.	1094.75	{1022.58, 1134.44}	131.447	0.364148	newly	313.	360.99	{335., 385.}	15.3898	0.0614824	Reff	0.632595	0.680586	{0.653796, 0.702791}	7.58636	ar	6867.	9212.56	{9041., 9384.}	34.157
20	R0	{1.53809, 10.5848}	{1.61852, 19.9038}	{{1.53966, 19.5352}, {1.67257, 20.}}	{5.32662, 88.0418}	inf	613.	915.392	{838.341, 1008.01}	49.3298	0.172907	newly	373.	311.15	{279., 345.}	16.5818	0.0802412	Reff	0.804882	0.820098	{0.775492, 0.862534}	3.40594	ar	6108.	7933.81	{7596., 8227.}	29.8921
21	R0	{1.59925, 6.74479}	{2.11102, 15.4157}	{{1.7535, 11.243}, {2.6037, 20.}}	{32.0006, 128.557}	inf	243.	1206.58	{819.274, 1657.79}	396.536	0.6789	newly	202.	493.81	{444., 541.}	144.46	0.386796	Reff	0.649775	0.619223	{0.518804, 0.704031}	10.2626	ar	6411.	9347.4	{8439., 10302.}	45.8025
22	R0	{1.38779, 4.40977}	{1.77057, 16.8597}	{{1.57472, 11.5929}, {1.91233, 20.}}	{27.5817, 282.327}	inf	176.	1170.38	{584.566, 1479.99}	564.989	0.802529	newly	221.	453.15	{337., 520.}	105.045	0.307117	Reff	0.751211	0.800679	{0.746155, 0.862161}	7.75703	ar	5154.	8304.67	{7243., 8917.}	61.1306
23	R0	{1.53657, 7.50352}	{1.81616, 17.1503}	{{1.60488, 13.4631}, {2.1064, 20.}}	{18.3356, 128.564}	inf	330.	1063.88	{715.336, 1411.11}	222.388	0.490181	newly	276.	398.73	{321., 462.}	44.9601	0.157413	Reff	0.757222	0.726153	{0.684355, 0.785629}	5.96351	ar	5928.	8395.47	{7626., 9224.}	41.624
24	R0	{1.71131, 6.24526}	{2.18128, 18.7146}	{{2.14624, 18.0184}, {2.25252, 20.}}	{27.4624, 199.661}	inf	308.	1619.34	{1486.99, 1889.39}	425.759	0.718931	newly	276.	559.15	{513., 616.}	102.591	0.305582	Reff	0.599814	0.594632	{0.570188, 0.627952}	3.55231	ar	6924.	9983.77	{9807., 10214.}	44.1908
25	R0	{1.4412, 3.07729}	{2.80056, 19.9015}	{{2.6487, 20.}, {2.90719, 20.}}	{94.3216, 546.721}	inf	56.	1976.21	{1849.14, 2151.34}	3428.95	1.54684	newly	93.	631.76	{580., 697.}	579.312	0.831092	Reff	0.624328	0.46746	{0.434648, 0.54336}	25.1258	ar	5810.	10557.7	{10351., 10725.}	81.7165
26	R0	{1.4159, 4.93858}	{1.80269, 14.3858}	{{1.62633, 10.3659}, {2.0822, 19.1398}}	{27.3179, 191.294}	inf	215.	1104.96	{790.147, 1391.97}	413.934	0.701837	newly	264.	506.15	{463., 548.}	91.7235	0.281605	Reff	0.740657	0.802728	{0.71283, 0.851566}	9.61527	ar	5255.	9071.18	{8320., 10022.}	72.62
27	R0	{1.57465, 7.05414}	{2.22401, 16.4922}	{{1.74653, 10.9115}, {2.672, 20.}}	{41.2384, 133.794}	inf	430.	1551.28	{915.371, 2008.83}	260.762	0.53643	newly	370.	602.31	{524., 667.}	62.7865	0.209902	Reff	0.69946	0.68157	{0.589508, 0.777046}	9.43089	ar	6359.	9603.77	{8517., 10425.}	51.0264
28	R0	{1.41422, 4.62402}	{2.20902, 17.3259}	{{1.75018, 12.4761}, {2.51175, 20.}}	{56.2008, 274.692}	inf	181.	1607.93	{1038.13, 1951.38}	788.36	0.939526	newly	219.	596.57	{543., 655.}	172.406	0.434075	Reff	0.681654	0.648138	{0.544757, 0.753897}	11.2468	ar	5485.	10147.4	{9132., 10754.}	85.0035
29	R0	{1.41901, 3.63017}	{2.84758, 18.7473}	{{2.47387, 16.8497}, {3., 20.}}	{100.673, 416.431}	inf	109.	1669.97	{1451.15, 1869.94}	1432.08	1.18161	newly	157.	548.94	{516., 589.}	249.643	0.54299	Reff	0.594707	0.394759	{0.347891, 0.462089}	33.884	ar	5979.	10751.5	{10400., 10996.}	79.8217
30	R0	{1.60719, 4.72984}	{2.90585, 19.3271}	{{2.49014, 16.4858}, {3., 20.}}	{80.8031, 308.622}	inf	159.	1958.2	{1628.78, 2166.15}	1131.57	1.08822	newly	176.	632.22	{562., 698.}	259.216	0.553963	Reff	0.595464	0.390141	{0.340078, 0.475683}	34.4811	ar	6479.	10822.9	{10499., 11005.}	67.0454
31	R0	{1.38175, 6.26468}	{1.69908, 12.525}	{{1.54942, 9.63715}, {1.83339, 15.8437}}	{22.9657, 99.931}	inf	255.	822.742	{550.634, 1084.25}	222.644	0.490664	newly	236.	424.45	{356., 484.}	79.8517	0.252053	Reff	0.70939	0.800987	{0.731334, 0.848191}	13.098	ar	5397.	8599.19	{7894., 9211.}	59.3328
32	R0	{1.4947, 8.8847}	{1.62296, 19.3826}	{{1.554, 18.2445}, {1.68916, 20.}}	{8.7045, 118.157}	inf	294.	833.909	{644.672, 944.83}	183.642	0.447798	newly	208.	286.6	{242., 318.}	37.8462	0.137009	Reff	0.787707	0.774016	{0.721139, 0.829966}	4.56565	ar	5407.	7857.12	{7423., 8168.}	45.3139
33	R0	{1.55891, 8.28978}	{1.73103, 19.8629}	{{1.62669, 19.4279}, {1.85237, 20.}}	{11.0414, 139.607}	inf	374.	1002.99	{909.365, 1056.73}	168.178	0.427689	newly	292.	338.79	{305., 368.}	16.2089	0.0642855	Reff	0.720061	0.755764	{0.670879, 0.809799}	7.45684	ar	6239.	8526.49	{8173., 8917.}	36.6644
34	R0	{1.60764, 10.4729}	{1.75531, 19.6877}	{{1.65041, 19.5717}, {1.89642, 20.}}	{9.57315, 87.9868}	inf	407.	977.571	{889.973, 1080.96}	140.19	0.377057	newly	229.	327.51	{291., 363.}	43.0175	0.153613	Reff	0.67971	0.719915	{0.644596, 0.765871}	7.73874	ar	6495.	8716.06	{8348., 9125.}	34.1965
35	R0	{1.48527, 5.74485}	{2.19592, 17.8998}	{{1.65474, 10.8625}, {2.41157, 20.}}	{47.8468, 211.581}	inf	235.	1398.27	{668.334, 1665.37}	495.007	0.756399	newly	239.	499.05	{401., 559.}	108.808	0.316782	Reff	0.703869	0.662407	{0.604743, 0.755555}	9.80764	ar	5811.	9684.77	{8196., 10225.}	66.6627
36	R0	{1.61457, 9.93364}	{1.88655, 19.6131}	{{1.77389, 19.3256}, {1.96674, 20.}}	{17.0218, 97.4409}	inf	545.	1305.23	{1215.72, 1455.69}	139.491	0.375321	newly	332.	439.46	{384., 483.}	32.3855	0.120086	Reff	0.74577	0.743109	{0.674944, 0.80079}	4.75437	ar	6338.	8968.69	{8606., 9229.}	41.5066
37	R0	{1.47831, 7.08139}	{1.79923, 16.4986}	{{1.60135, 11.5801}, {2.11709, 20.}}	{21.7087, 132.985}	inf	340.	1084.54	{569.449, 1482.86}	218.981	0.483511	newly	278.	425.78	{320., 495.}	53.1583	0.180133	Reff	0.759556	0.760538	{0.691025, 0.836927}	5.83247	ar	5530.	8370.49	{7491., 9218.}	51.3651
38	R0	{1.73445, 9.8858}	{2.13717, 19.7047}	{{2.01703, 18.6277}, {2.26493, 20.}}	{23.2603, 99.3231}	inf	569.	1568.34	{1467.11, 1712.54}	175.631	0.438482	newly	367.	520.26	{469., 573.}	41.7602	0.150275	Reff	0.688056	0.653726	{0.601483, 0.713595}	6.47624	ar	6842.	9471.35	{9307., 9675.}	38.4296
39	R0	{1.66621, 11.4824}	{1.81486, 19.8862}	{{1.6971, 19.7187}, {1.92277, 20.}}	{9.15813, 73.1883}	inf	533.	1152.1	{1071.83, 1254.66}	116.154	0.333079	newly	292.	384.37	{348., 426.}	31.6404	0.1179	Reff	0.756459	0.732049	{0.65833, 0.783137}	5.76715	ar	6473.	8759.82	{8370., 9110.}	35.3286
40	R0	{1.60694, 9.95741}	{1.87819, 19.8317}	{{1.78497, 20.}, {1.95477, 20.}}	{16.9816, 99.1657}	inf	510.	1274.78	{1179.83, 1372.23}	149.957	0.395605	newly	314.	422.54	{382., 461.}	34.6242	0.127835	Reff	0.690181	0.711044	{0.664129, 0.756319}	5.36246	ar	6625.	9243.85	{8922., 9490.}	39.5298
41	R0	{1.55128, 4.71911}	{2.68753, 18.254}	{{2.25006, 16.7289}, {3., 20.}}	{73.2459, 286.811}	inf	233.	1780.88	{1547.9, 2187.88}	664.326	0.879671	newly	259.	621.36	{564., 714.}	139.907	0.378261	Reff	0.655881	0.515539	{0.398218, 0.634501}	22.4636	ar	6141.	10723.7	{10238., 11120.}	74.6253
42	R0	{1.60063, 6.81118}	{1.88302, 19.9444}	{{1.81312, 20.}, {1.93876, 20.}}	{17.7371, 192.819}	inf	295.	1212.23	{1167.53, 1278.85}	310.925	0.613109	newly	240.	399.17	{374., 433.}	66.3208	0.220204	Reff	0.619924	0.716787	{0.675365, 0.756135}	15.625	ar	6528.	9497.3	{9290., 9718.}	45.4856
43	R0	{1.56986, 5.44652}	{2.42599, 17.1194}	{{1.93457, 11.9789}, {2.61446, 19.2095}}	{54.5354, 214.317}	inf	202.	1460.19	{1000.18, 1778.81}	622.869	0.852843	newly	196.	542.85	{495., 610.}	176.964	0.441076	Reff	0.625118	0.584424	{0.533722, 0.708874}	9.82599	ar	6312.	10652.5	{9563., 11068.}	68.7662
44	R0	{1.47523, 8.07785}	{1.78734, 18.2932}	{{1.57106, 14.359}, {1.95416, 20.}}	{21.1565, 126.462}	inf	323.	1092.05	{768.777, 1320.73}	238.097	0.518085	newly	237.	388.63	{329., 441.}	63.9789	0.211762	Reff	0.760776	0.746108	{0.682406, 0.808323}	5.6133	ar	5692.	8561.2	{7742., 9129.}	50.4076
45	R0	{1.6417, 7.69429}	{2.25493, 19.0876}	{{2.09148, 17.2723}, {2.40969, 20.}}	{37.3537, 148.075}	inf	363.	1441.59	{1339.35, 1578.07}	297.133	0.597096	newly	273.	482.66	{432., 542.}	76.7985	0.245895	Reff	0.605951	0.532612	{0.472851, 0.579402}	12.9541	ar	6735.	9954.34	{9606., 10262.}	47.8001
46	R0	{1.38865, 5.83473}	{1.71704, 16.7116}	{{1.5484, 12.054}, {1.85789, 20.}}	{23.6546, 186.417}	inf	305.	1015.44	{551.362, 1374.16}	233.03	0.501302	newly	319.	396.5	{299., 471.}	27.3981	0.103629	Reff	0.760702	0.812951	{0.75914, 0.879058}	7.33063	ar	5233.	8077.47	{7226., 8752.}	54.3564
47	R0	{1.45289, 6.87903}	{1.71124, 15.4009}	{{1.53302, 11.4484}, {1.98783, 20.}}	{17.8038, 123.882}	inf	301.	991.043	{615.598, 1519.45}	229.25	0.493279	newly	259.	417.3	{336., 519.}	61.1197	0.201748	Reff	0.779475	0.82541	{0.73615, 0.882214}	7.70664	ar	5379.	8149.4	{7384., 9157.}	51.504
48	R0	{1.69416, 9.4831}	{2.01124, 20.}	{{1.90601, 20.}, {2.16889, 20.}}	{18.7162, 110.901}	inf	573.	1423.48	{1336.33, 1517.03}	148.425	0.394642	newly	377.	469.27	{427., 514.}	24.4748	0.0940495	Reff	0.683255	0.706644	{0.642421, 0.772322}	6.76648	ar	6925.	9601.41	{9354., 9883.}	38.6485
49	R0	{1.47843, 7.16286}	{1.81369, 17.303}	{{1.65459, 13.8646}, {2.1044, 20.}}	{22.6768, 141.565}	inf	252.	1128.26	{771.125, 1452.48}	347.722	0.637251	newly	214.	423.48	{358., 485.}	97.8879	0.293065	Reff	0.752669	0.759321	{0.69992, 0.831539}	5.3969	ar	5449.	8427.54	{7797., 9192.}	54.6621
50	R0	{1.68843, 5.72427}	{2.05982, 10.2396}	{{1.92409, 8.77103}, {2.21037, 11.4892}}	{21.9962, 78.881}	inf	170.	667.119	{515.238, 778.609}	292.423	0.581172	newly	156.	397.44	{345., 446.}	154.769	0.40346	Reff	0.581326	0.653599	{0.609323, 0.694513}	12.8819	ar	6800.	9581.32	{9137., 10024.}	[illegible]
